# Supplementary figures and images for: Chromosome-Level Genome Assembly of Papilio elwesi Leech, 1889 (Lepidoptera: Papilionidae)
Source: Insects. 2023 Mar 21;14(3):304. doi: 10.3390/insects14030304 (PMC10058580; doi:10.3390/insects14030304)

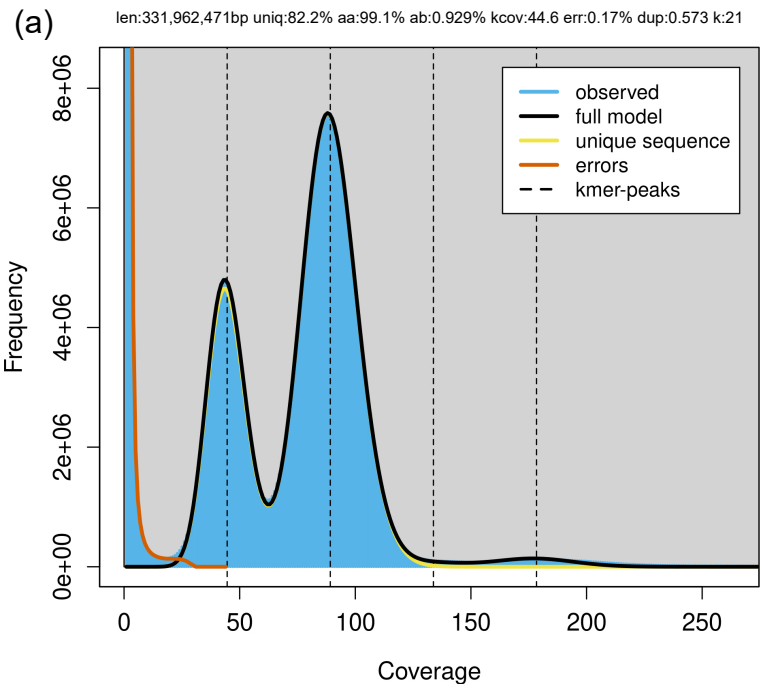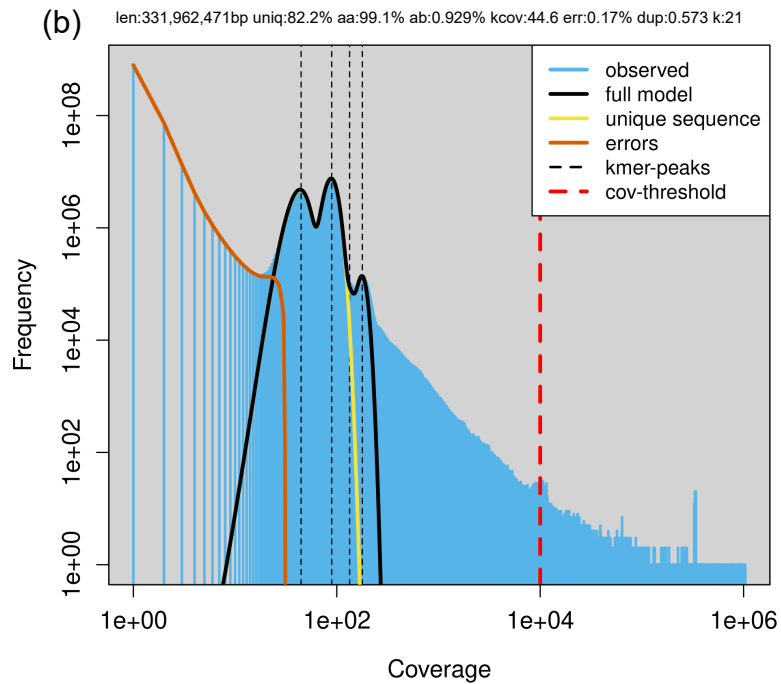

Supplement: Supplementary file 1 [file insects-14-00304-s001.zip › Figure S1.pdf]

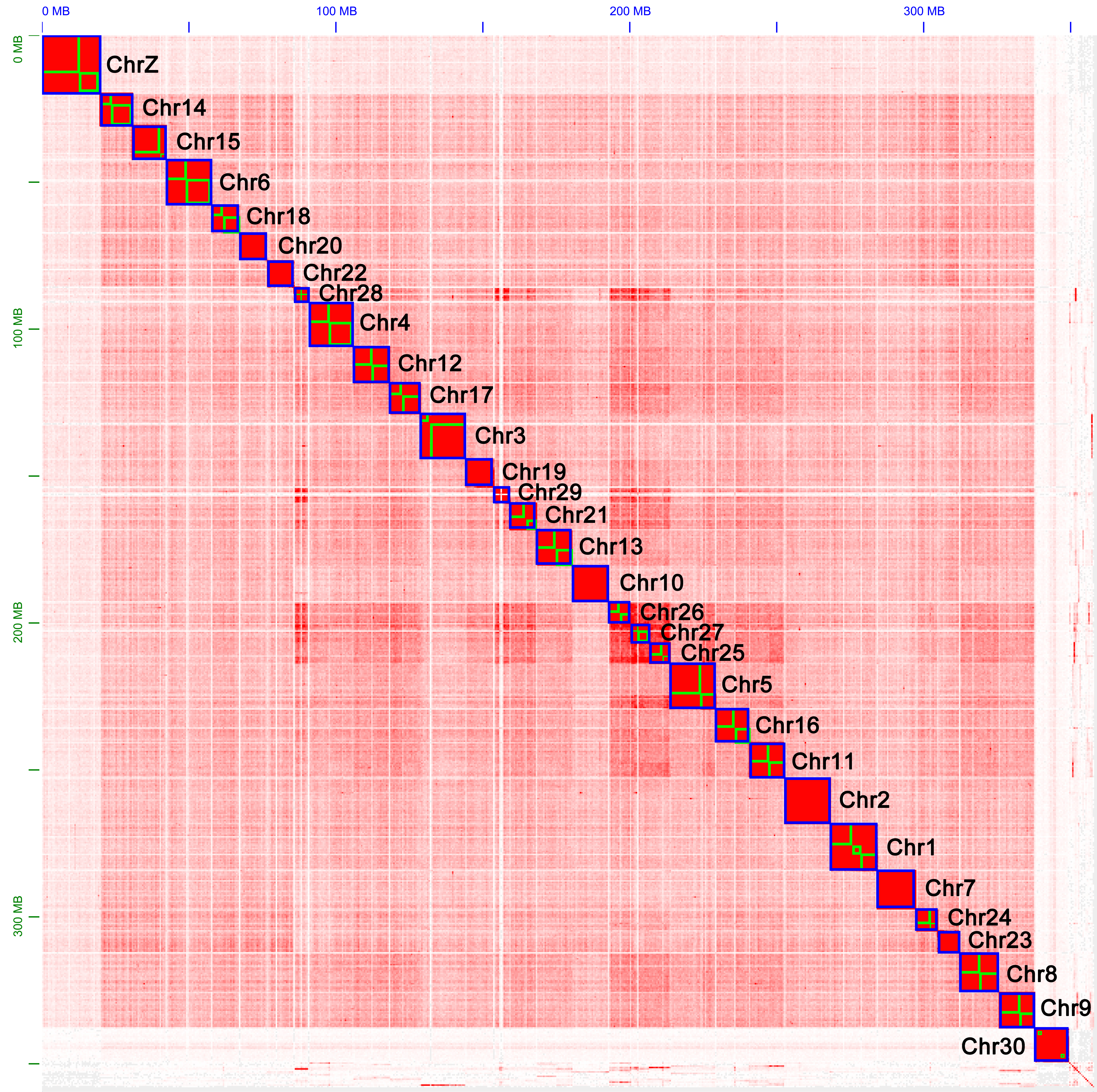

Supplement: Supplementary file 1 [file insects-14-00304-s001.zip › Figure S2.pdf]

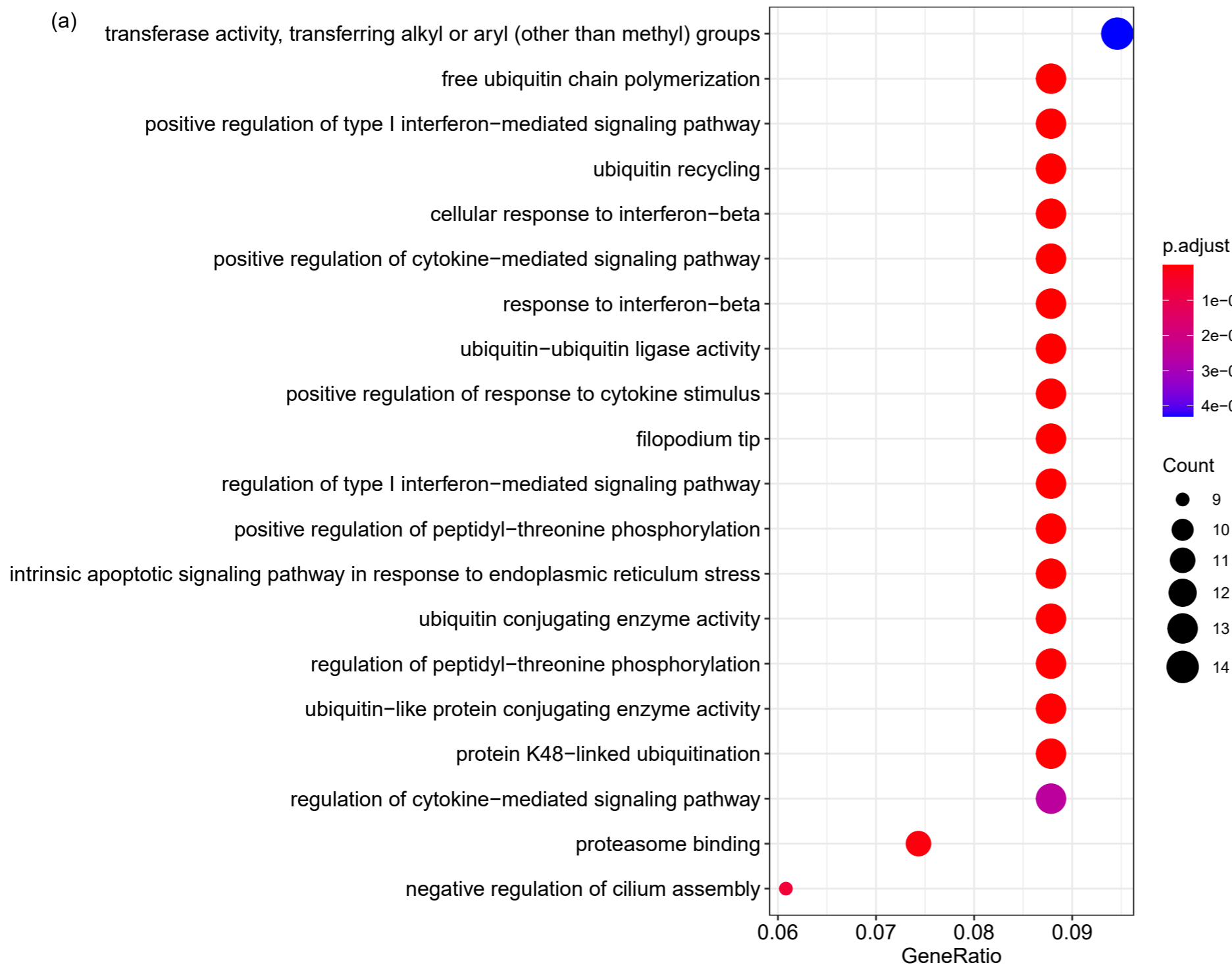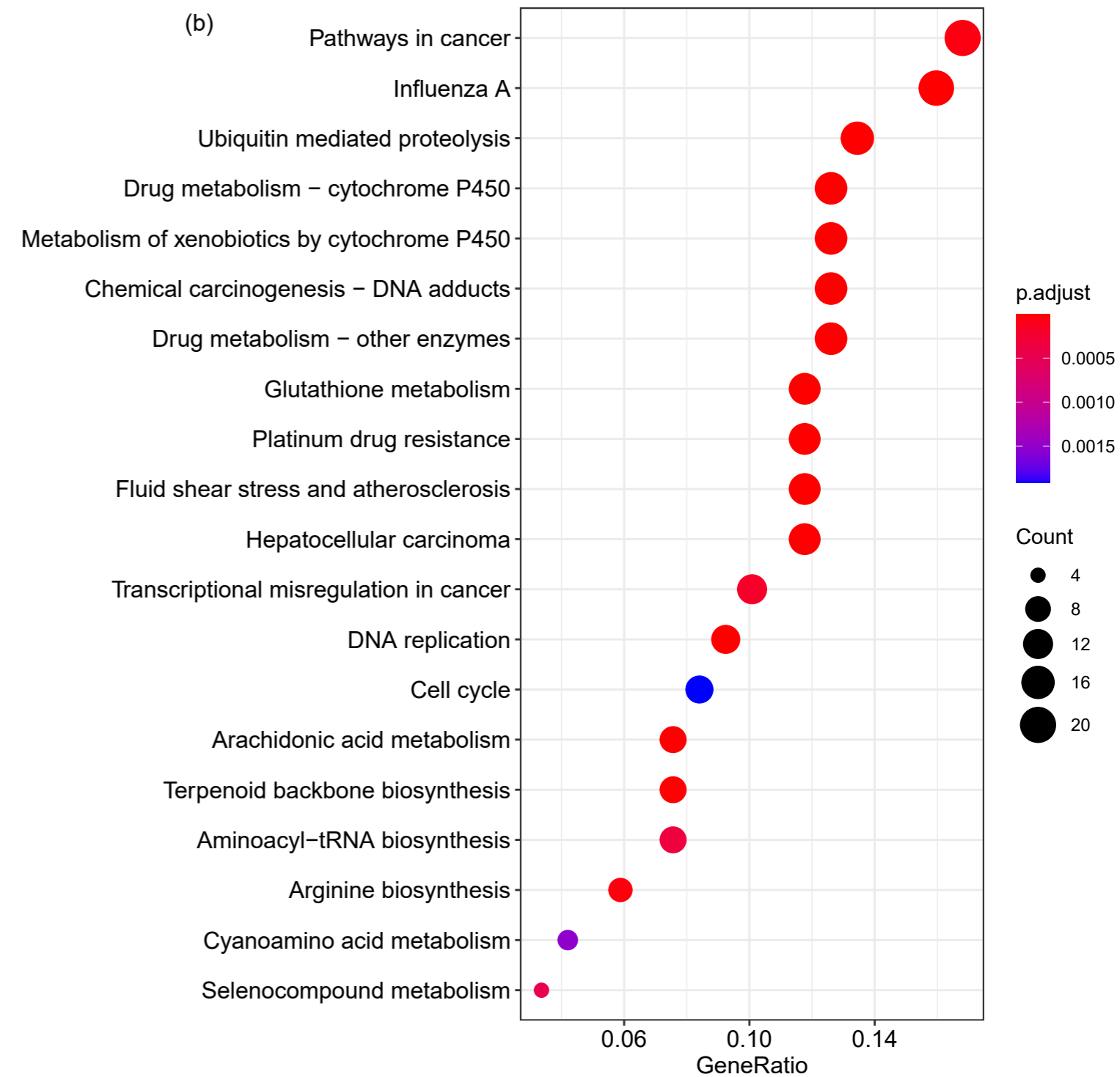

Supplement: Supplementary file 1 [file insects-14-00304-s001.zip › Figure S3.pdf]
